# Enhanced Photocatalytic Degradation of Methyl Orange Dye under the Daylight Irradiation over CN-TiO_2_ Modified with OMS-2

**DOI:** 10.3390/ma7128024

**Published:** 2014-12-12

**Authors:** Mohamed Elfatih Hassan, Jing Chen, Guanglong Liu, Duanwei Zhu, Jianbo Cai

**Affiliations:** Laboratory of Eco-Environment Engineering Research, Key Laboratory of Arable Land Conservation, Ministry of Agriculture, College of Resources & Environment, Huazhong Agricultural University, Wuhan 430070, China; E-Mails: mshassan@163.com (M.E.H.); chenjing20122@163.com (J.C.); zhudw@mail.hzau.edu.cn (D.Z.); Jbcai@mail.hzau.edu.cn (J.C.)

**Keywords:** CN-codoped, OMS-2, daylight, photocatalysis, TiO_2_

## Abstract

In this study, CN-TiO_2_ was modified with cryptomelane octahedral molecular sieves (OMS-2) by the sol-gel method based on the self-assembly technique to enhance its photocatalytic activity under the daylight irradiation. The synthesized samples were characterized by X-ray diffraction (XRD), UV-vis spectroscopy, Fourier transform infrared spectroscopy (FT-IR) and porosimeter analysis. The results showed that the addition of OMS-2 in the sol lead to higher Brunauer-Emmett-Teller (BET) surface area, pore volume, porosity of particle after heat treatment and the specific surface area, porosity, crystallite size and pore size distribution could be controlled by adjusting the calcination temperature. Compared to the CN-TiO_2_-400 sample, CN-TiO_2_/OMS-2-400 exhibited greater red shift in absorption edge of samples in visible region due to the OMS-2 coated. The enhancement of photocatalytic activity of CN-TiO_2_/OMS-2 composite photocatalyst was subsequently evaluated for the degradation of the methyl orange dye under the daylight irradiation in water. The results showed that the methyl orange dye degradation rate reach to 37.8% for the CN-TiO_2_/OMS-2-400 sample under the daylight irradiation for 5 h, which was higher than that of reference sample. The enhancement in daylight photocatalytic activities of the CN-TiO_2_/OMS samples could be attributed to the synergistic effects of OMS-2 coated, larger surface area and red shift in adsorption edge of the prepared sample.

## 1. Introduction

Today, TiO_2_ has been the most widely studied material for the photocatalytic application [1,2,3,4]. However, the practical applications of TiO_2_ are limited by its large band gap (3.2 eV), which can only active under the UV light irradiation [5,6,7]. Therefore, several strategies have been developed to shift the optical sensitivity of TiO_2_ from UV to the visible-light region for the efficient use of solar energy, such as element doping, metal deposition, surface sensitization, and coupling of composite semiconductors [8]. In element doping, besides traditional single element doping [9], co-doped TiO_2_ with two or more elements was confirmed to be an effective way for higher visible light photocatalytic activity. For example, N–F [10,11], N–S [12], B–N [13], and C–N [14,15,16,17,18] co-doped TiO_2_ were reported to significantly improve the photocatalytic efficiency under visible light illumination. In our previous paper, carbon and nitrogen co-doped TiO_2_ exhibited higher photocatalytic activity than that of reference sample [19]. Unfortunately, nonmetal elements doping can also increase electron-hole recombination rates and lead to an overall decrease in performance despite enhanced light absorption [20].

Combining other semiconductor or support provide a promising way to address this problem. With this approach, the constituents are selected so that efficient charge transfer between them, further to reduce the rate of electron-hole recombination. Examples of composite photocatalysts with improve performance include SnO_2_/TiO_2_ [21], ZnO/TiO_2_ [22,23], and CdS/TiO_2_ [24,25,26], among others. However, paying attention to the efficient charge transfer, has neglected the effect of specific surface area, which affects the adsorption rate and photocatalytic efficiency because pre-adsorption pollutant molecules on TiO_2_ particles facilitate degradation. Therefore, another strategy involves the TiO_2_ over support materials with high specific surface area, such as activated carbon, zeolite, silica, and Al_2_O_3_ [27,28,29,30]. However, the substantial materials decrease photocatalytic activity due to inefficient light harvesting. Thus, the development of new active supports is important for substantial materials to achieve enhanced photocatalytic activity of TiO_2_.

Due to its octahedral molecular sieves structure, high redox potential, and large specific surface area [31], cryptomelane has been reported to have excellent catalytic properties [32], and has been extensively tested in toluene heterogeneous oxidation [33] and 2,4-dichlorophenoxyacetic acid photocatalytic degradation [34]. From our previous work, there should be Mn^4+^/Mn^3+^ in the Mn oxide, which could benefit the charge transfer from the TiO_2_ to octahedral molecular sieves (OMS-2) [35]. Therefore, in the present study, OMS-2 was employed to modify the CN-TiO_2_, enhance its photocatalytic activity in methyl orange dye degradation under the daylight irradiation and the probable photocatalytic mechanism under daylight irradiation was proposed.

## 2. Results and Discussion

### 2.1. Physical Properties of CN-TiO_2_/OMS-2 and Reference Samples

**Figure 1:**
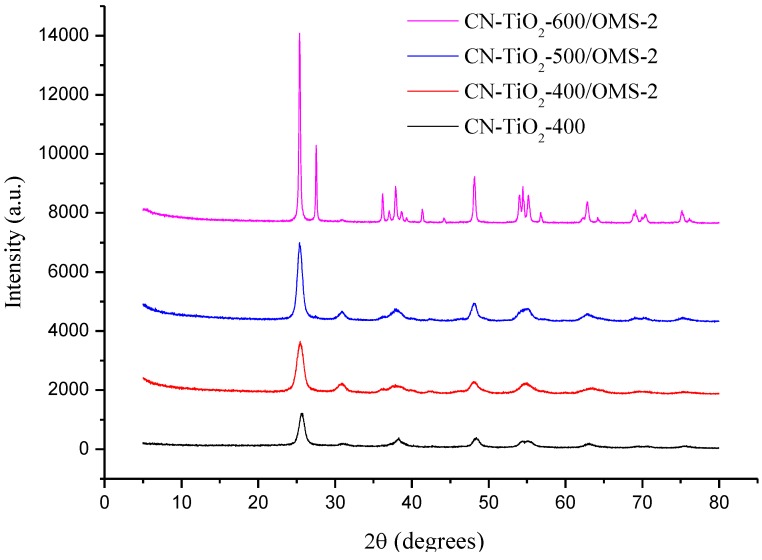
XRD patterns of as-prepared reference and CN-TiO_2_/OMS-2 nanoparticles calcined at different temperature.

The XRD patterns of the reference and samples at different calcinations temperatures were shown in Figure 1, Well-defined, broad, diffraction peaks corresponding to the anatase TiO_2_ phase were identified for the CN-TiO_2_ and CN-TiO_2_/OMS-2-400 sample. However, the rutile TiO_2_ phase appeared for the CN-TiO_2_/OMS-2 sample when the calcinations temperature increased to 600 °C. The major peak at 25.3° of anatase is observed in all CN-TiO_2_/OMS-2 samples and this peak became narrow with the calcinations temperature increasing, which pointed to the increase of the TiO_2_ crystallite size and crystallinity. The average crystallite size of 16.5, 17.0, 21.8 and 22.9 nm were thus determined by Scherrer formula for the most intense (101) diffraction peak of anatase [36] for CN-TiO_2_/OMS-2-400, CN-TiO_2_/OMS-2-500 and CN-TiO_2_/OMS-2-600, respectively (Table 1). For the CN-TiO_2_/OMS-2 samples, it was found that XRD analysis did not reveal the presence of peaks corresponding to OMS-2 on composite catalysts, as lower concentration of OMS-2 might escape from the detection of XRD, which was similar with other report [37].

**Figure 2:**
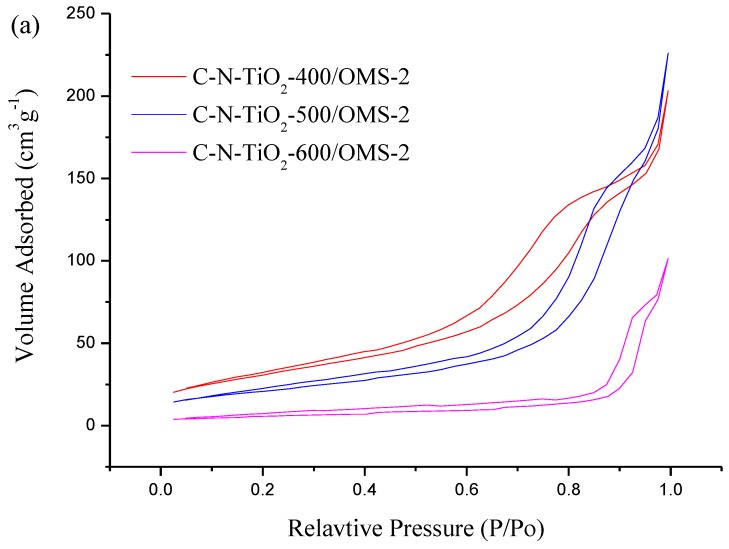

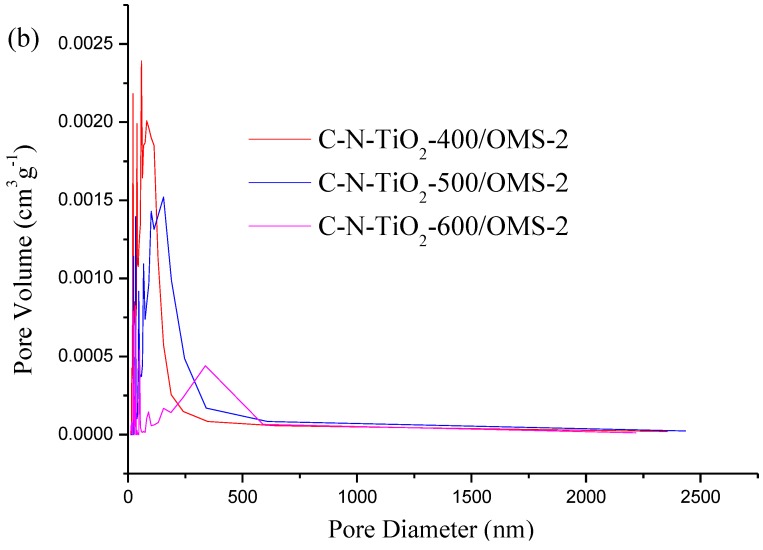
(**a**) Nitrogen adsorption-desoprtion isotherms and (**b**) pore size distribution of CN-TiO_2_/OMS-2 samples with different calcination temperature.

The N_2_ adsorption-desorption isotherms and pore size distribution curves of the CN-TiO_2_/OMS-2 samples calcined at different temperature are shown in Figure 2. The pore size distribution was calculated from the desorption branch of nitrogen isotherms by barret-joyner-Halenda (BJH) method using the Halsey equation. It can be seen that the N_2_ adsorption-desorption isotherms of CN-TiO_2_/OMS-2 samples vary with increasing calcination temperature as shown in Figure 2a. The CN-TiO_2_/OMS-2 with different calcination temperature shows the significant hysteresis loop in the adsorption-desorption isotherms, which indicates that the synthesized CN-codoped TiO_2_ samples are typical mesoporous materials [38]. The pore size distribution of the resulting samples showed considerable difference as displayed in Figure 2b. All samples showed unimodal pore size distribution and the pore size distribution of CN-TiO_2_/OMS-2-400 was very narrow implying good homogeneity of the pores. With increasing of calcination temperature, the pore size distribution of samples became broad, which due to sintering and growth of crystallites, though the material retained its highly porous characteristics [39]. With increasing of calcination temperature, the bigger crystallites aggregation could form bigger pores. Therefore, the diameter of pore became bigger while the volume of pore became smaller with increasing calcination temperature [40]. The Brunauer-Emmett-Teller (BET) specific surface areas of CN-TiO_2_/OMS-2-400 sample and reference material (calcinations at 400 °C) are recorded at 114.7 m^2^·g^−1^ and 40.1 m^2^·g^−1^ (summarized in Table 1), respectively. The mesoporous structure and high BET surface area of CN-TiO_2_/OMS-2 are expected to enhance adsorption of organic compounds onto the particle surface. It is noteworthy that the loading of OMS-2 on the TiO_2_ particles has greatly increased the specific area of the material. This phenomenon can be attributed to the presence of the OMS-2, which acts as a dispersing template to downsize the TiO_2_ nanostructures during the synthesis process. Therefore, the adsorption capacity and photocatalytic activity of the synthesized particles could be enhanced since adsorption processes are predominantly driven by surface reaction on the materials.

**Table 1:** Structural characteristics of CN-TiO_2_/OMS-2 with calcination temperature and reference material.

Samples	*S*_BET_(m^2^·g^−1^) ^a^	Pore volume (cm^3^·g^−1^)	Crystal phase	Crystal size (nm) ^b^
CN-TiO_2_-400	40.1	0.074	Anatase	16.5
CN-TiO_2_/OMS-2-400	114.7	0.252	Anatase	17.0
CN-TiO_2_/OMS-2-500	79.5	0.278	Anatase	21.8
CN-TiO_2_/OMS-2-600	21.9	0.187	Anatase+Rutile	22.9

a. Determined by N_2_ porosimetry by converting the adsorbed gas amount to liquid volume at the relative pressure of 0.99; b. Based on XRD using Scherrer Formula *D* = 0.9λ/(*B* × cosθ), where λ = 0.154 nm and *B* = full width at half maximum (FWHM) of the highest peak.

**Figure 3:**
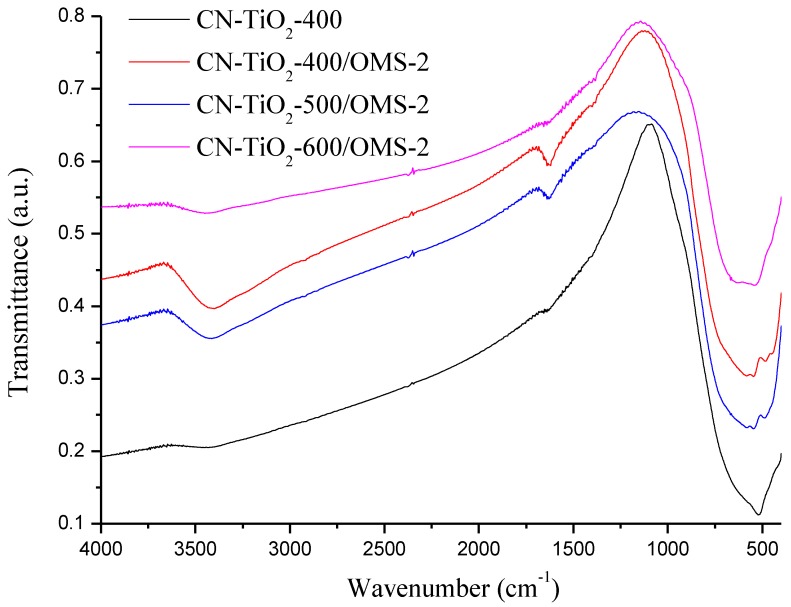
FT-IR spectra of CN-TiO_2_/OMS-2 samples and reference.

To give additional evidence and further to confirm the doping of OMS-2, Fourier transform infrared spectroscopy (FT-IR) characterizations were preformed. The infrared spectroscopy of CN-TiO_2_/OMS-2 samples and reference material are presented in Figure 3. The characteristic IR absorption peaks at 3136, 2330, 1640, 1400 and 530 cm^−1^ were observed in all samples. The bands at 1640 cm^−1^ and the wide bands at 3136 cm^−1^ are resultant from O–H bending of adsorbed water molecules, which has been reported that OH bonded group on the catalyst surface could benefit the photoctalysis efficiency of TiO_2_ materials. It noteworthy that the intensity of OH bonded group in CN-TiO_2_/OMS-2 was stronger than that of reference sample, which may be due to the OH bonded group in the doped OMS-2. This would enhance the photocataly activity of CN-TiO_2_/OMS-2 sample. The bands at 2330 cm^−1^ are assigned to the stretching vibrations of the C=O bonds, which should be attributed to the T80 used in the sol. The bands at 1400 cm^−1^ are assigned to the stretching vibrations of the N–H bonds in NH_3_, which evidences the presence of NH_3_ in Ti complex [16,17]. The NH_3_ in the Ti complex is regarded as nitrogen source for the N-doped TiO_2_. With the calcination temperature increasing, the N–H bands at 1400 cm^−1^ disappear, which evidences the decomposition of NH_3_. The characteristic IR absorption peaks at 530 and 470 cm^−1^ are spectral features of OMS-2. Compared with the IR absorption spectrum of the CN-TiO_2_/OMS-2-400 and reference sample, it shows both the characteristic IR absorption of CN-TiO_2_/OMS-2-400 and that of the reference sample around 530 cm^−1^. It proved that the synthesized powder was the composite of OMS-2 and CN-TiO_2_.

**Figure 4:**
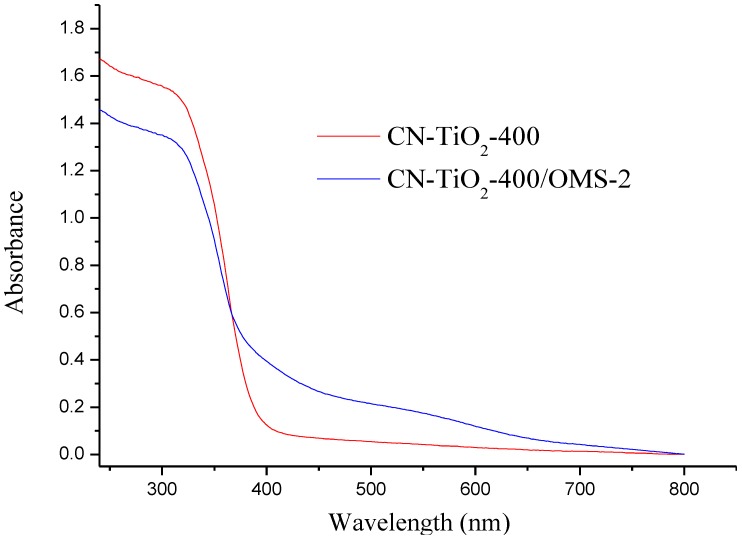
Optical properties of CN-codoped TiO_2_/OMS-2 sample compared to reference.

Figure 4a shows the UV-vis absorption spectra of the CN-TiO_2_/OMS-2-400 and reference samples. It has been reported that pure TiO_2_ only absorbed ultraviolet radiation of less than 400 nm. Due to the carbon and nitrogen co-doping, the reference sample showed obvious absorption in the visible light region of 400–700 nm. Compared to the reference sample, the CN-TiO_2_/OMS-2-400 exhibited stronger absorption in the visible light region. It indicated that the presence of carbon and nitrogen co-doping or synergistic effect between OMS-2 and TiO_2_ support that aid the photocatalytic activity in the visible-light region. In the present study, OMS-2 may interact with TiO_2_ and CN-codoping also increased the visible light absorption ability of the composite.

### 2.2. Photocatalytic Activity of CN-TiO_2_/OMS-2 Samples

**Figure 5:**
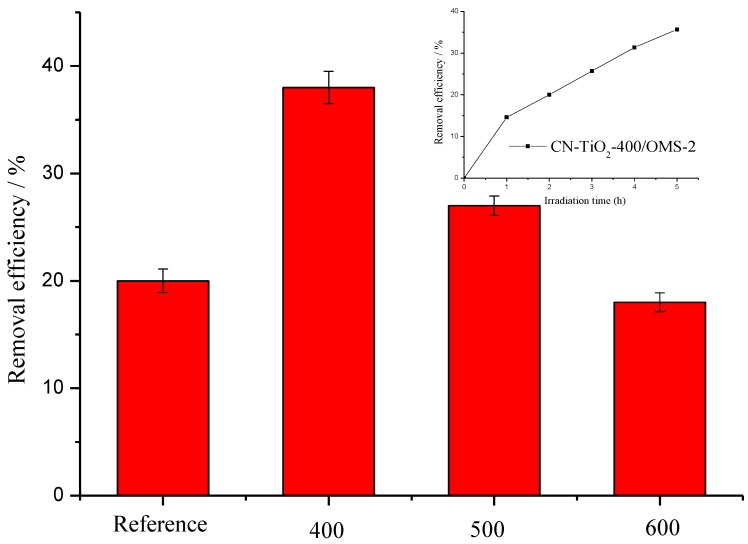
The dependence of the removal efficiency (*R* (%)) of photocatalytic oxidation of methyl orange under daylight irradiation on calcination temperature (Insertion is methyl degradation by CN-TiO_2_/OMS-2).

Figure 5 shows the photocatalytic degradation of methyl orange dye by the CN-TiO2/OMS-2 samples with various calcination temperature and reference sample under the daylight irradiation. It can be seen that the 20% methyl orange dye was degraded by the reference sample, which should be contributed to the C and N co-doped on the TiO_2_. When the CN-TiO_2_/OMS-2 samples were added, the methyl orange dye degradation rate increased and CN-TiO_2_/OMS-2-400 exhibited highest photocatalytic activity in the methyl orange dye degradation under the daylight irradiation. However, the removal efficiencies steadily decreased with the calcination temperature increased. This result further confirmed that the sample calcined at 400 °C possessed the highest photocatalytic activity. From our results, the photocatalytic activity of samples was extremely related with the heat treatment, which were agreement with other reports. For the CN-TiO_2_/OMS-2 sample, the photocatalytic activity of samples decreased with the calcination temperature increase, which may be due to the C and N removed in the higher temperature. This will reduce the e^−^ and h^+^ generated on the surface of samples, further reducing the photocatalytic activity of samples.

### 2.3. Photocatalytic Mechanism

**Figure 6:**
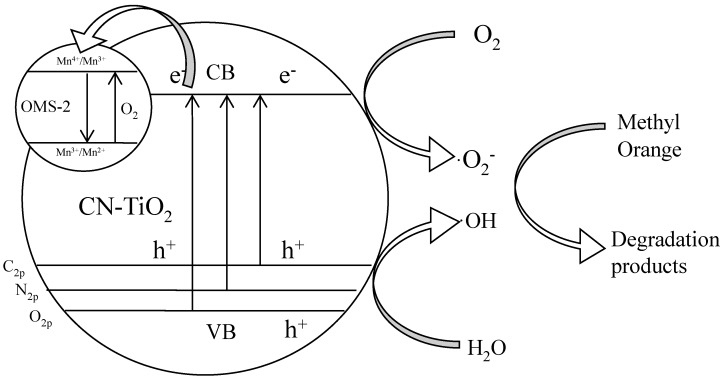
Possible mechanism for the photocatalytic activity of CN-TiO_2_/OMS-2 samples to methyl orange degradation.

The band structure of bare CN-TiO_2_/OMS-2 is shown in Figure 6. The O 2p orbitals contribute to make up a valence band (VB) while Ti 3d contributes for a conduction band (CB). Since the distance between VB (O 2p) and CB (Ti 3d) of pure TiO_2_ is large (3.31 eV) (Figure 6), absorption of visible-light is negligible and hence TiO_2_ showed low photocatalytic activity in the degradation of methyl orange. On the other hand, the new absorption band appeared below the conduction band edge of TiO_2_ in CN-TiO_2_ (Figure 6) due to C and N doped is responsible for visible light absorption in CN-TiO_2_. For the CN-TiO_2_/OMS-2 samples, the OMS-2 nanoparticle was coated on the surface of CN-TiO_2_ support. It has been reported that Mn^4+^/Mn^3+^ existed in the Mn oxide due to the average oxidation state (AOS) of 3.75 in OMS-2, which could benefit the charge transfer from the TiO_2_ to OMS-2 [35]. When visible-light was illuminated on CN-TiO_2_/OMS-2, the electrons excited from the valence band to conduction band leaving holes in the valence band of TiO_2_. The excited electrons in the conduction band are transferred to Mn^4+^/Mn^3+^ resulting the reduction of Mn^4+^/Mn^3+^ to Mn^3+^/Mn^2+^. Hence the holes with strong oxidation power generated in the VB of CN-TiO_2_/OMS-2 and the electrons in the conduction band are effectively separated upon visible-light irradiation. This circulatory system will inhibit the electron-hole recombination, increase the generated rate of •OH, and improve the photocatalytic activity. For the methyl orange dye degradation, the dye could transfer to the excited state of dye under the light irradiation, which rapidly reacted with •OH and resulted in the degradation [41].

## 3. Experimental Section

### 3.1. Chemicals

T80 (Guoyao Chemical Co., Shanghai, China), isopropyl alcohol (i–PrOH, 99.8%, Guoyao Chemical Co.), titanium tetraisopropoxide (TTIP, 97%, Sigma-Aldrich, St. Louis, MO, USA), acetic acid (AcOH, Guoyao Chemical Co., Shanghai, China), HNO_3_ (Sinopharm Chemical Reagent Co., Shanghai, China), and methyl orange (Sinopharm Chemical Reagent Co., Shanghai, China) were used as received.

### 3.2. Synthesis of Daylight-Activated CN-TiO_2_/OMS-2 Samples

CN-TiO_2_/OMS-2 sample was prepared by the self-assembly surfactant-based sol-gel method under mild conditions as follow. A nonionic surfactant T80 was employed as the pore directing agent and carbon precursor in the modified sol-gel solution. T80 (4 g) was dissolved in isopropanol (20 mL) and then acetic acid (3 mL) was added for the *in-situ* formation of water. Before adding the Ti precursor, anhydrous ethylenediamine (3 mL) was induced in the solution as a nitrogen source. Then, titanium (IV) isopropoxide (6 mL) was added dropwise under vigorous stirring, and 20 mL acetic acid was added. The transparent, homogeneous and stable solution was obtained after stirring 24 h at room temperature. Then, 0.0468 g OMS-2 prepared as Liu *et al.* [35] reported was added to the solution and aged at room temperature for 10 h. To synthesize particles, the solution was dried at 120 °C for 6 h and then calcined at 400, 500 and 600 °C for 3 h. For comparison, CN-TiO_2_ and TiO_2_ samples were prepared though the same method, without adding the corresponding dopants and calcined at 400 °C for 3 h.

### 3.3. Characterization of Synthesized CN-TiO_2_/OMS-2 Samples

To study the crystal structure and crystallinity of the CN-TiO_2_/OMS-2 and references samples, X-ray diffraction (XRD) analysis was performed on X’Pert PRO (D8 Advance, Bruker Co., Bremen, Germany) using Cu Kα (λ = 1.5406 Å) radiation. The Brunauer-Emmett-Teller (BET) surface area, pore volume, porosity, Barret-Joyner-Halenda (BJH) pore size and distribution (based on nitrogen adsorption and desorption isotherms) were determined by Tristar 300 (Micromeritics, Micromeritics Instrument Corporation, Norcross, GA, USA) porosimeter analyzer. All the test powders were collected form the films and purged with nitrogen gas for 2 h at 150 °C using Flow prep 060 (Micromeritics). Fourier transform infrared (FT-IR) spectroscopy was carried out using Thermo Scientific Nicolet 6700 spectrometer (Waltham, MA, USA) to detect the presence of carbon group on the samples. Measurement range was 4000–400 cm^−1^, with a 4 cm^−1^ resolution, 0.475 cm^−1^/s scan speed and 32 scans. The technique applied was attenuated total reflectance (ATR) with an Avantar multibounce HATR accessory with ZnSe crystal at 45°. To investigate the light absorption and optical band gap of the synthesized TiO_2_ nanoparticles, the UV-vis absorption spectra were obtained with a UV-vis spectrophotometer (UV-2450 PC, Shimadzu Co., Kyoto, Japan) mounted with an integrating sphere accessory (ISR1200) using BaSO_4_ as reference standard.

### 3.4. Photocatalytic Evaluation with Methyl orange under Daylight

After synthesis and characterization, Photocatalytic activity CN-TiO_2_/OMS-2 samples were evaluated by the degradation of methyl orange dye under simulated daylight using two fluorescent lamps (20W, Shanghai Yaming Co. Ltd., Shanghai, China) as light source and the light intensity on the surface of reactor was 2 mW/cm^2^ measured using a UV intensity meter (UV-I, Beijing Shida Ltd., Beijing, China). Methyl orange is a pH indicator and was examined in the red form. To this end, the pH of the sample was adjusted with nitric acid to acidic condition. The degradation rate of the dyes was monitored by recording the variations of the particular absorption-band maximum (λ_max_ = 517 nm, as seen in Figure S1) in the absorbance spectrum using a UV2450 UV-vis spectroscopy. (Shimadzu, Kyoto, Japan). Before each experimental, a particles suspension (0.5 g/L) solution was prepared and dispersed using an ultrasonicator (2510R-DH, Bransonic, Beijing, China) for 24 h. 10 mL methyl orange solution (500 mg/L) was transferred to a 50 mL particles suspension placed in reactor to achieve an initial concentration of 100 mg/L. Finally, 50 μL HNO_3_ (0.05 mol·L^−1^) was added into the solution. During the irradiation, the reactor was sealed with parafilm and continuously mixed to minimize mass transfer limitations. A 2 mL sample was withdrawn at time 0, 1, 2, 3, 4 and 5 h. The photocatalyst was immediately removed from the samples after centrifugation and the concentration of methyl orange dye in the samples was determined by UV-vis spectroscopy.

## 4. Conclusions

The daylight-active carbon and nitrogen co-doped TiO_2_ combined with OMS-2 was successfully synthesized by the sol-gel method based on the self-assembly technique. The CN-TiO_2_/OMS-2 samples exhibited lager surface area, pore volume and porosity of particle than that of sample without of OMS-2 adding. It was also found that the specific surface area, porosity, crystallite size and pore size distribution of TiO_2_/OMS-2 samples could be controlled by adjusting the calcination temperature. The TiO_2_/OMS-2-400 sample exhibited the highest photocatalytic activity during the degradation of methyl orange dye among samples calcined at other temperature and reference sample under the daylight irradiation. The enhanced daylight activity of TiO_2_/OMS-2 could be attributed to the OMS-2 doping, synergistic effects of carbon, nitrogen and OMS-2 doping, large surface area, good crystallization and red shift in adsorption edge of the prepared sample.

## Supplementary Materials

Supplementary materials can be accessed at: http://www.mdpi.com/1996-1944/7/12/8024/s1.
