# Supplementary material for: Enhanced Photocatalytic Degradation of Methyl Orange Dye under the Daylight Irradiation over CN-TiO2 Modified with OMS-2
Source: Materials (Basel). 2014 Dec 12;7(12):8024–36. doi: 10.3390/ma7128024 (PMC5456436; doi:10.3390/ma7128024)

Supplementary Information

Figure S1. UV-vis spectra of methyl orange under neutral condition and acidic condition.

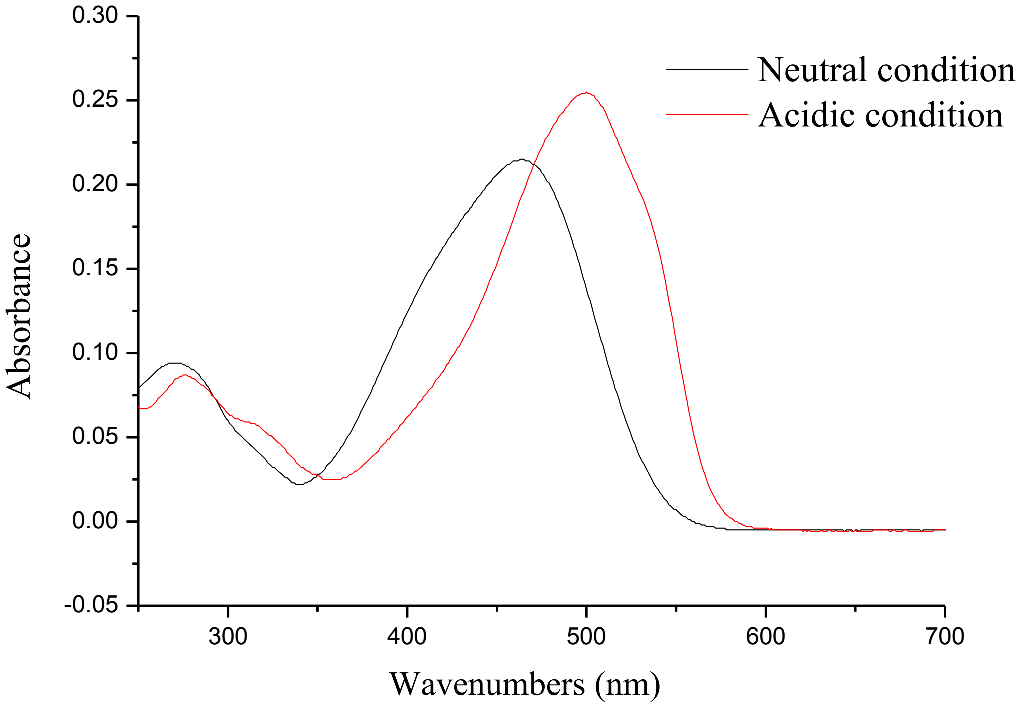

Supplement: Supplementary file 1 [file materials-07-08024-s001.pdf]
